# Relationship among Bone Mineral Density Reduction, Hearing Loss, and Balance Disorders in Osteoporotic Patients

**DOI:** 10.3389/fbioe.2013.00017

**Published:** 2013-11-25

**Authors:** Fatemeh Radaei, Shahriar Gharibzadeh

**Affiliations:** ^1^Neural and Cognitive Sciences Laboratory, Biomedical Engineering Faculty, Amirkabir University of Technology, Tehran, Iran

**Keywords:** osteoporotic patients, osteoporotic fractures, BMD loss, hearing loss, balance disorder, hormonal mechanism, vestibule, labyrinth

Osteoporosis is a disease that weakens bones and is known to be “silent,” but it does not mean that it should be ignored. According to some researches, osteoporosis causes more than 8.9 million fractures every year, resulting in an osteoporotic fracture every 3 s and by 2050 the world-wide occurrence of hip fracture is estimated to increase by 310% in men and 240% in women (Gullberg et al., 1997; Johnell and Kanis, 2006).

The main aspect of osteoporosis is losing bone mineral density (BMD). Mainly bones consist of minerals such as a matrix of hydroxyapatite (calcium phosphate) and other minerals which are embedded in a cross-linked collagen matrix. Normally, there is a delicate balance between bone formation and bone re-absorption, which if bone re-absorption becomes dominant osteopenia and osteoporosis may occur (Larsen, 2007). In another word, in osteoporotic patients due to reduction of bone formation in comparison with bone re-absorption, low bone mineral is stored and as a result BMD loss occurs (Bone et al., 2000; Marie, 2013).

On the other hand, according to some researches, it has been shown that there is a correlation between BMD loss in osteoporotic patients and conductive hearing loss; in those patients over a specific age, changing the structure of the ossicles or hormonal mechanism in hearing may correlate BMD loss with hearing loss (Shafer, 2006; Babich et al., 2009).

In fact, the hearing and balance systems are connected inside the inner ear and hearing loss can affect balance system. Basically, Balance is a complex that receives sensory information from a variety of organs (vision, auditory, and joints) and integrates it to inform the body of where it is. Furthermore, information from the vestibular system of the inner ear (semicircular canals, the saccule and the utricle) is sent to the brain. Semicircular canals in the inner ear are able to sense changes in movement of the body. Due to these changes, endolymph within the canals moves inner ear’s hair cells. As a result, position of the head is sensed by hair cells. Normally, the information coming from the ears perfectly matches the information coming from the eyes and the sensors in the joints and balance complex completes (Standring, 2004; Wedro, 2009). What is more, there are some evidences that show due to metabolic dysfunction in bones – osteoporosis – there is a correlation between hearing loss and balance disorders (Zatonski et al., 2012).

**Figure 1:**
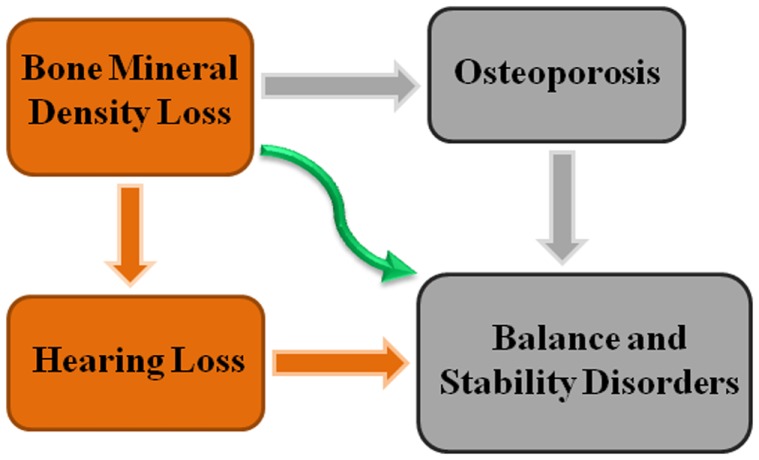
**Relationship between BMD loss and balance disorders in osteoporotic patients**.

In conclusion, we hypothesize that in osteoporotic patients, who have low BMD, hearing loss occurs and as a result balance problems happen. In spite of the fact that some researchers have shown that osteoporotic patients have balance disorders and hearing loss (Henkin et al., 1972; Matsuo et al., 2005), the possible relationship with BMD loss and hearing loss has not been clarified up to now (Figure 1). In fact, our methodology toward this study would be based on clinical records of patients. Basically, patients’ BMD score, history of falling (summary of their status of stability), and their auditory test score is needed to provide our hypothesis. Last but not least, it is proposed that using some specific earphones which intensify the sound, by compensating the hearing loss in patients may reduce risk of falls in osteoporotic patients who suffer from balance problems, but obviously earphones are not preventive enough for falling. Surely experimental researches and clinical trials are needed to validate our hypothesis.
